# Supplementary material for: Composition Characterization of Crossostephium chinense Leaf Essential Oil and Its Anti-Inflammatory Activity Mechanisms
Source: Plants (Basel). 2024 Sep 6;13(17):2506. doi: 10.3390/plants13172506 (PMC11397578; doi:10.3390/plants13172506)
Supplement: Supplementary file 1 [file plants-13-02506-s001.zip › plants-3192947-supplementary.pdf]

Supplementary Information

Composition Characterization of *Crossostephium chinense* Leaf Essential Oil and its Anti-inflammatory Activity Mechanism

Chia-Hsin Lin[1,2], Yu-Ting Chiang[1], Nai-Wen Tsao[3], Chung-Hsuan Wang[1], Yin-Shuan Sun[1], Sheng-Yang Wang[1,3,4]*

[1] Department of Forestry, National Chung Hsing University, Taichung 402202, Taiwan

[2] Department of Chinese Pharmaceutical Sciences and Chinese Medicine Resources,

China Medical University, Taichung 404328, Taiwan

[3] Special Crop and Metabolome Discipline Cluster, Academy Circle Economy, National Chung Hsing University, Taichung 402202, Taiwan

[4] Agricultural Biotechnology Research Center, Academia Sinica, Taipei City 115201, Taiwan

Correspondence

Sheng-Yang Wang,

Department of Forestry, National Chung-Hsing University, 250 Kuo-Kuang Road, Taichung,402, Taiwan, China

Fax: +886-4-22873628, Phone: +886-4-22840345 ext. 138.

E-mail: taiwanfir@dragon.nchu.edu.tw (S-Y. Wang)

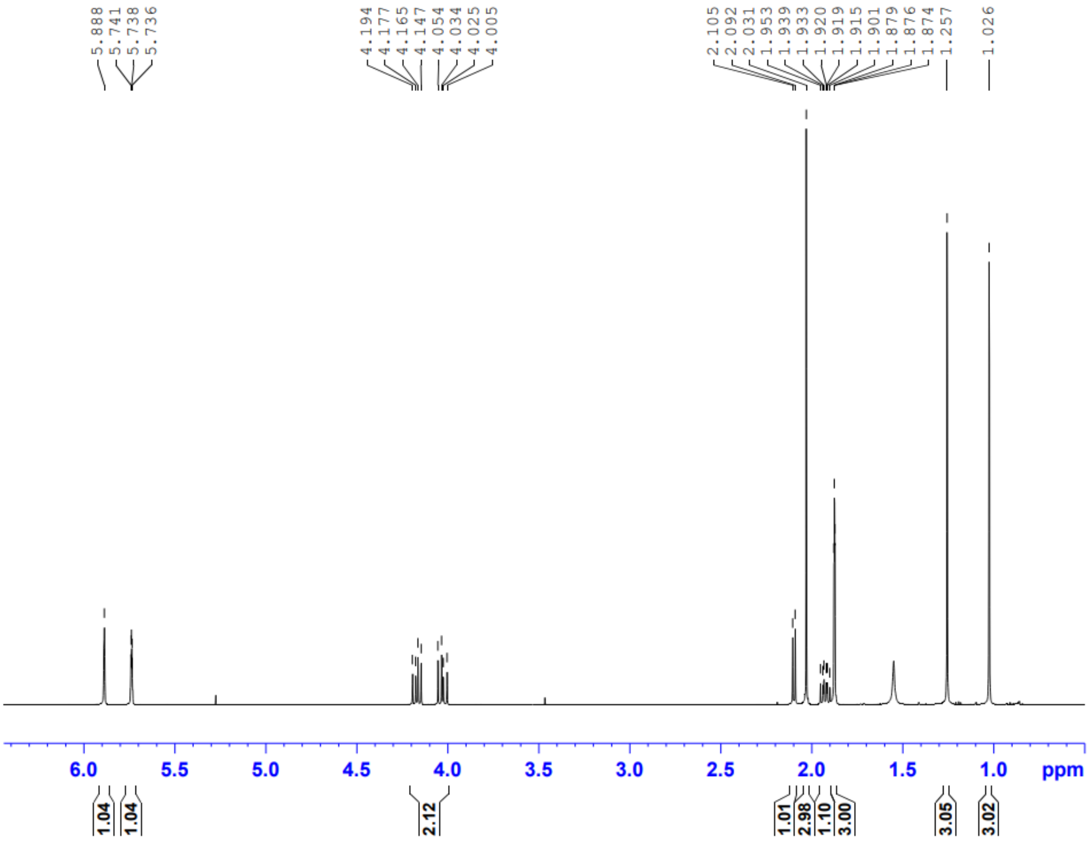

S1. ^{1}H-NMR spectrum of compound 1 ($CDCl_3$, 400 MHz)

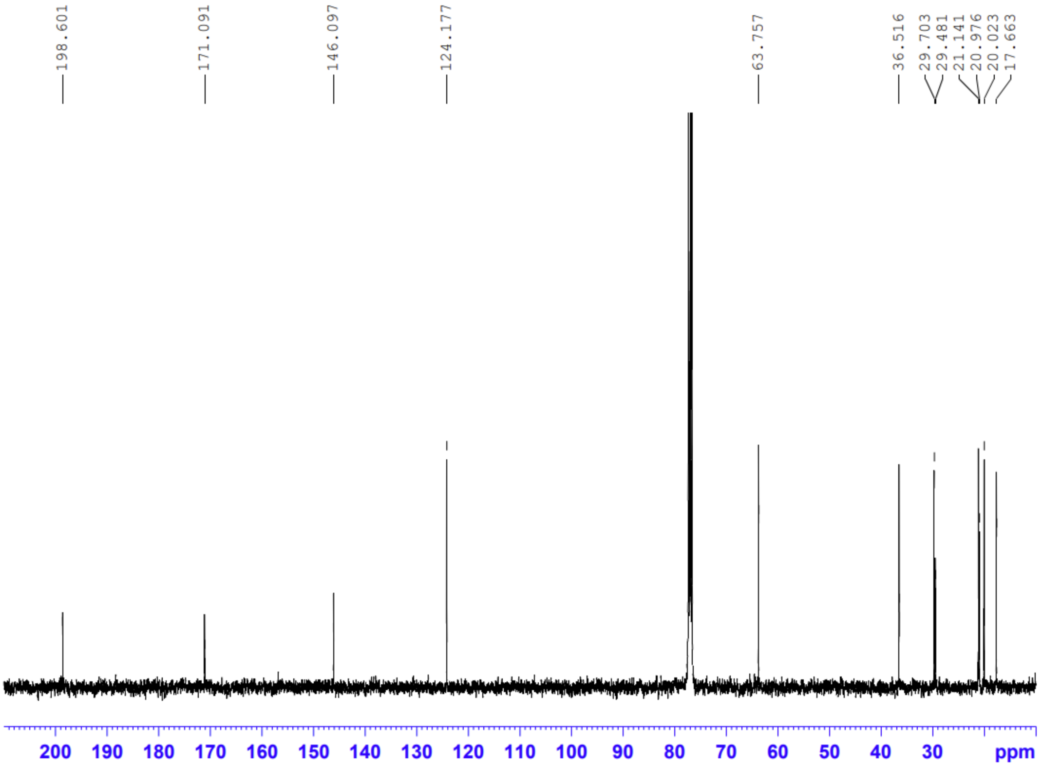

S2. ^{13}C-NMR spectrum of compound 1 ($CDCl_3$, 100 MHz)

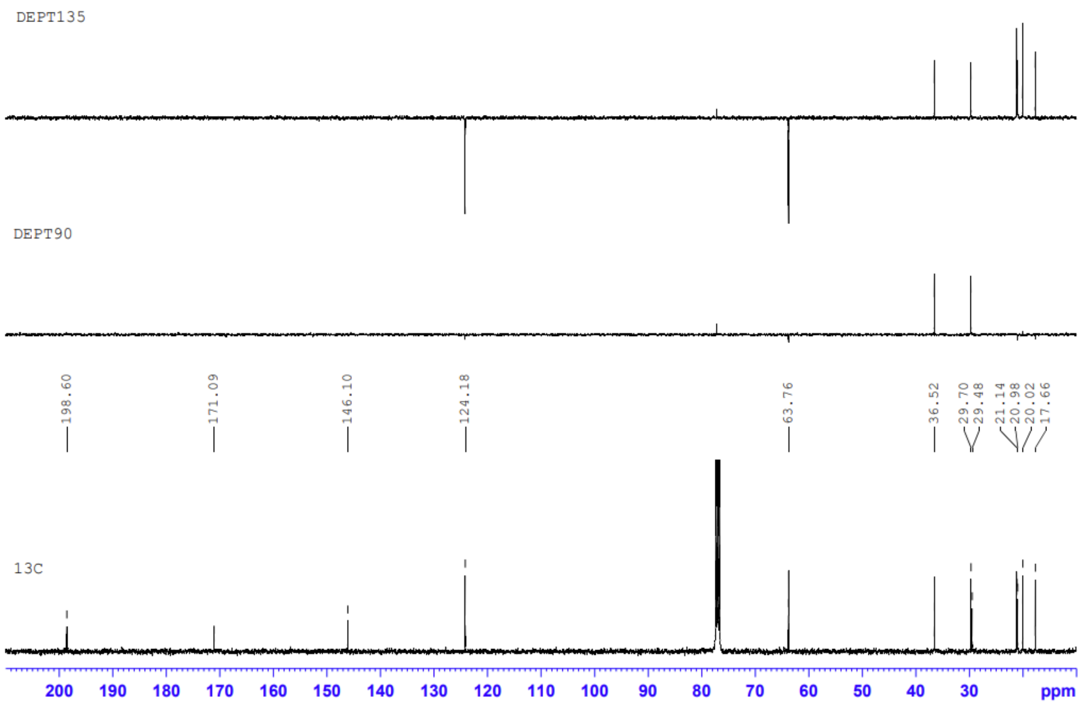

S3. DEPT NMR spectrum of coumpound 1 ($CDCl_3$, 100 MHz).

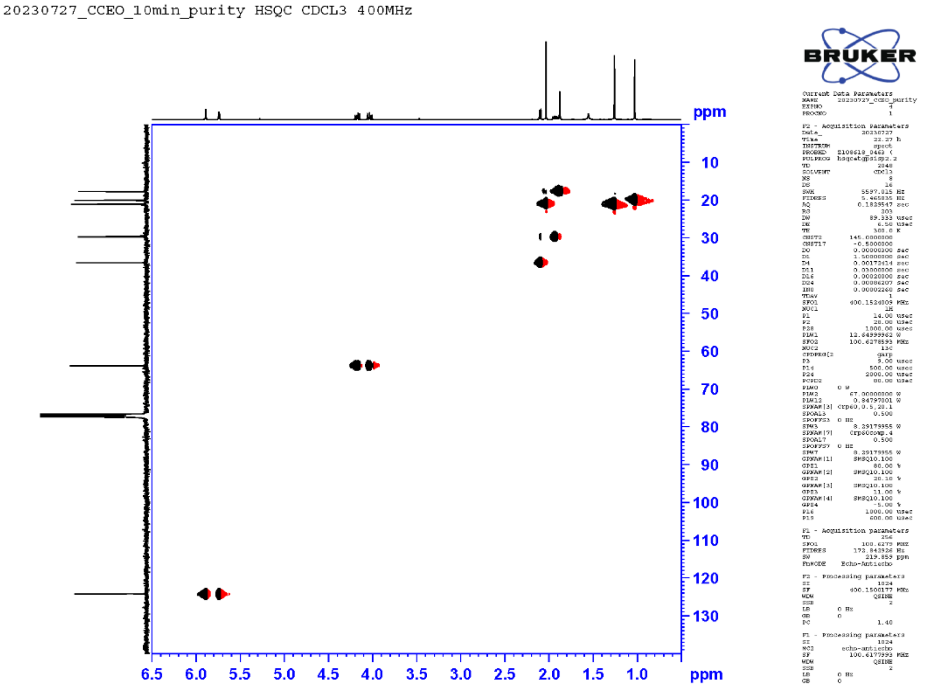

S4. HSQC spectrum of compound 1.

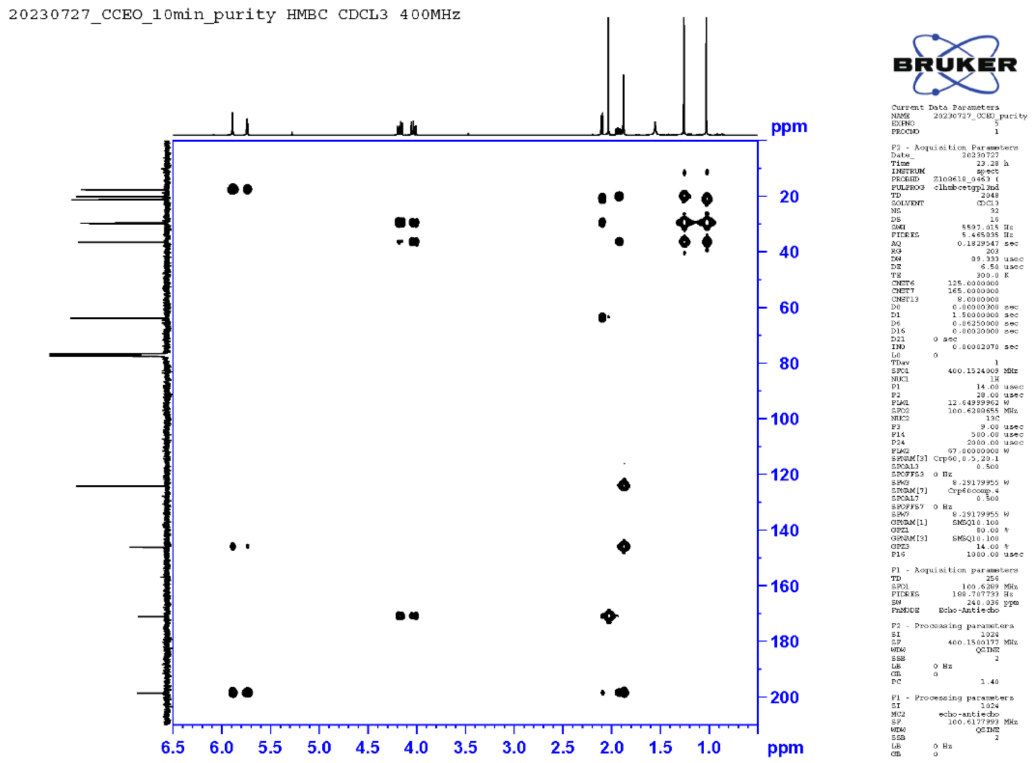

S5. HMBC spectrum of compound 1.

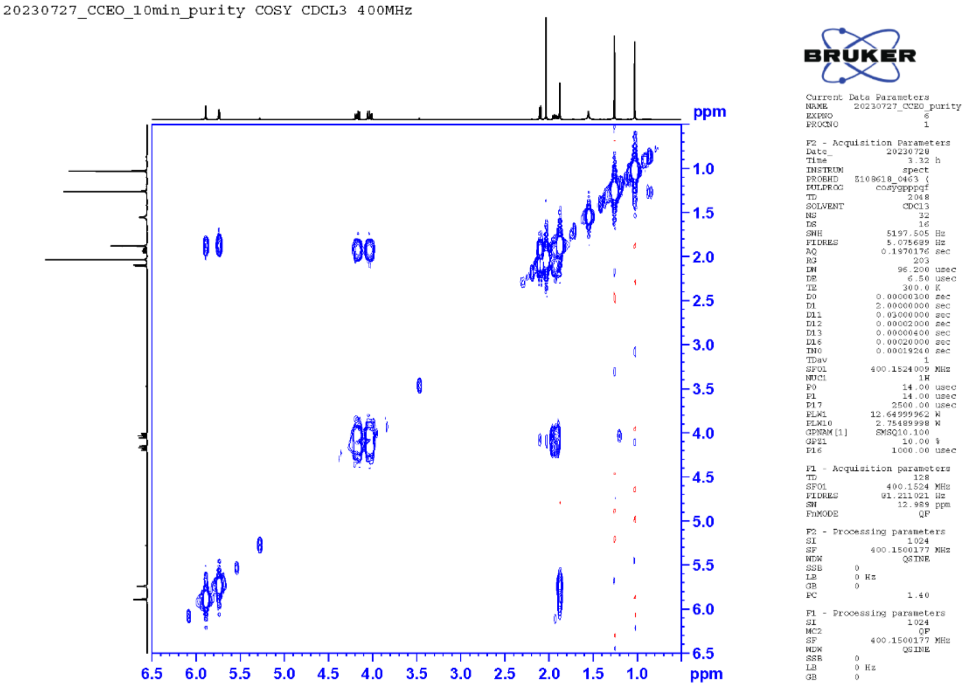

S6. COSY spectrum of compound 1.

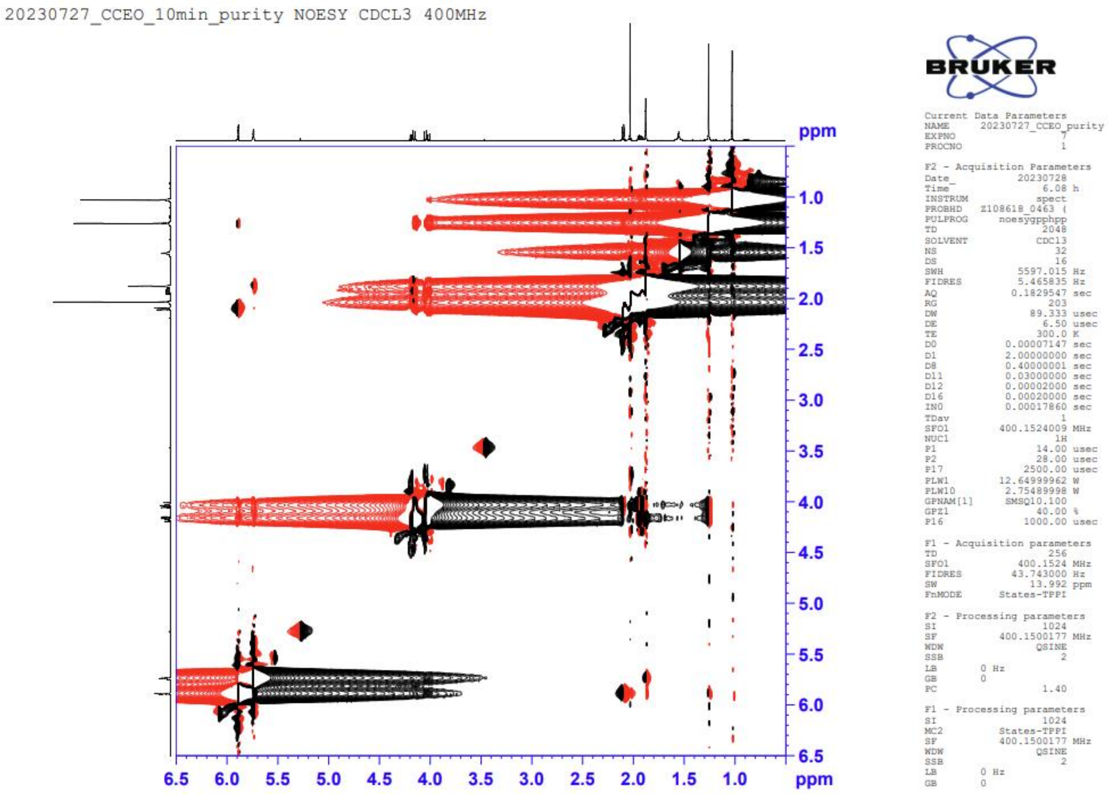

S7. NOSEY spectrum of coumpound 1.

```
LIST: hei1027_cc-2-c3                    24-Jun-24 REG   : 01:04.8     #9
Samp:                                              Start : 15:28:38   2653
Mode: EI +VE +LMR  ESCAN (EXP) UP HR NRM
Oper:                                              Inlet :
Limt: (  0)     .    .
    : (455) C30.H31.O4
Peak: 1000.00 mmu   R+D: -2.0 > 60.0
Data: +/189>260  (CMASS : converted; CMASS : converted; CMASS : conve

              799                  (mmu)
    Mass  Intensity     %RA   Flags Delta  R+D  Composition
212.1415     223462   83.83   #      -0.3  3.0  C12.H20.O3
```

S8. Analysis result of compound 2 by high-resolution mass spectrometry.

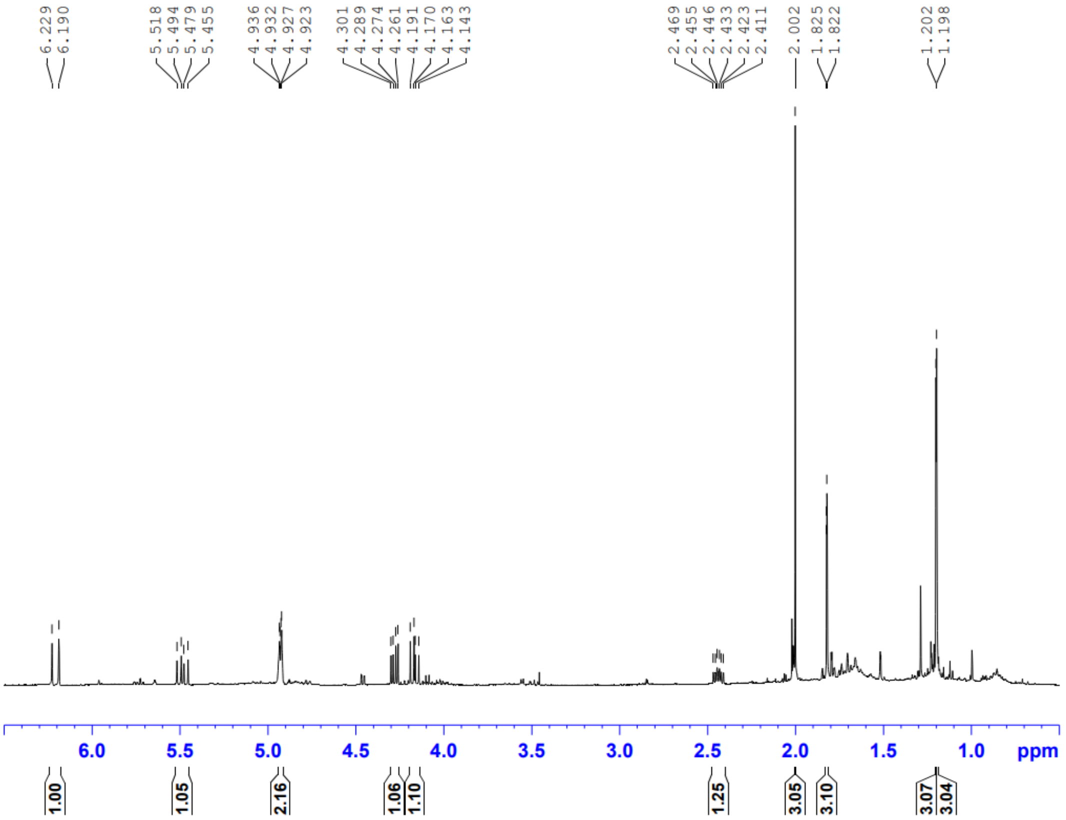

S9. ^{1}H-NMR spectrum of compound 2 ($CDCl_3$, 400 MHz)

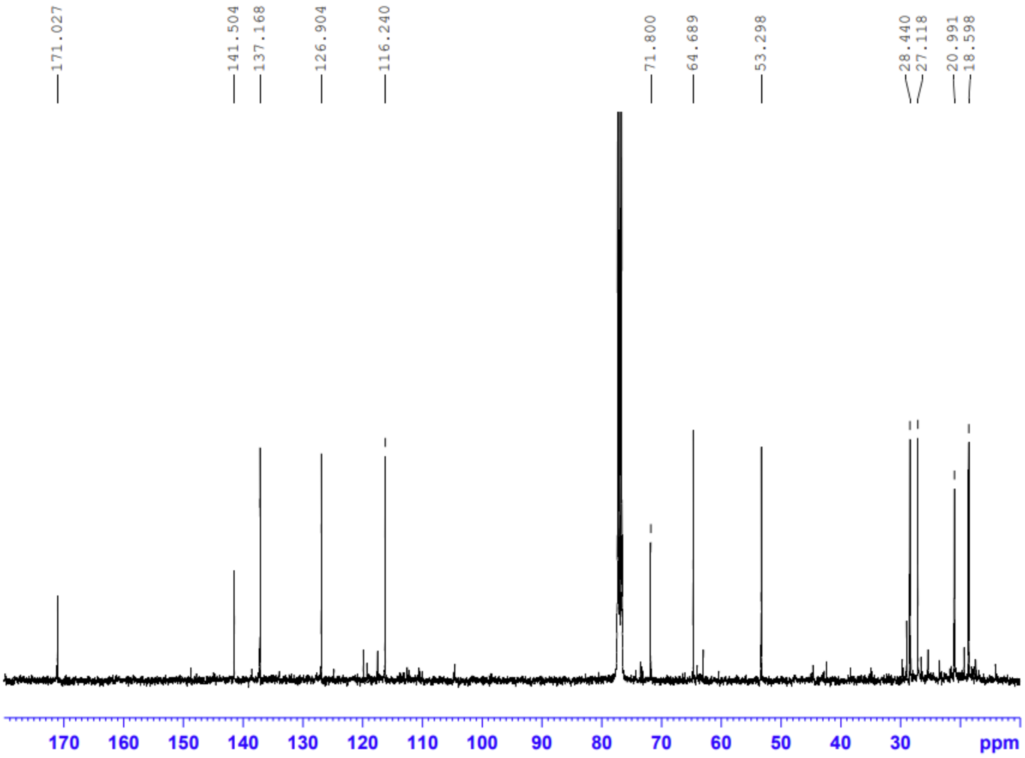

S10. ^{13}C-NMR spectrum of compound 2 ($CDCl_3$, 100 MHz)

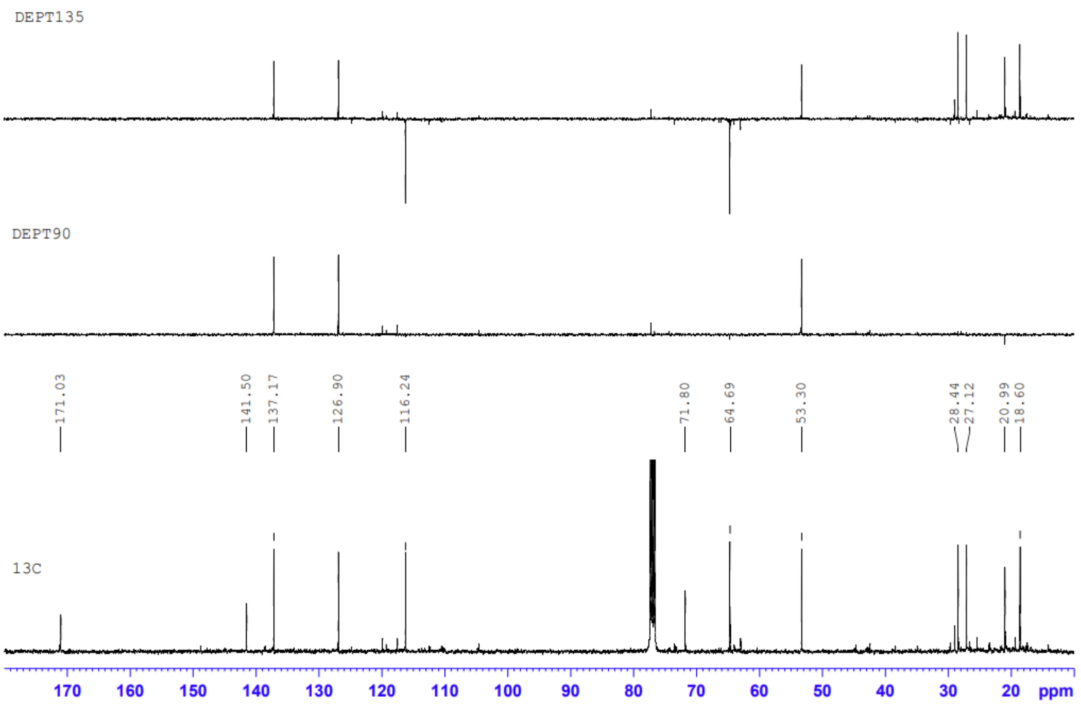

S11. DEPT NMR spectrum of coumpound 2 ($CDCl_3$, 100 MHz).

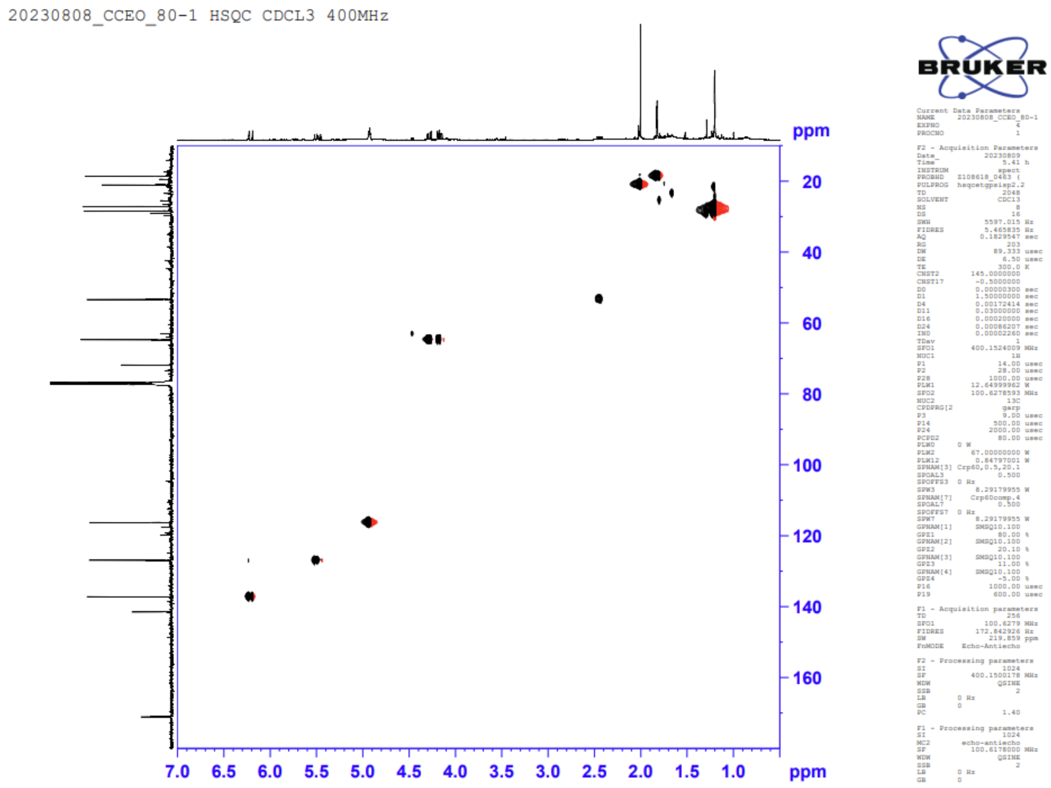

S12. HSQC spectrum of compound 2

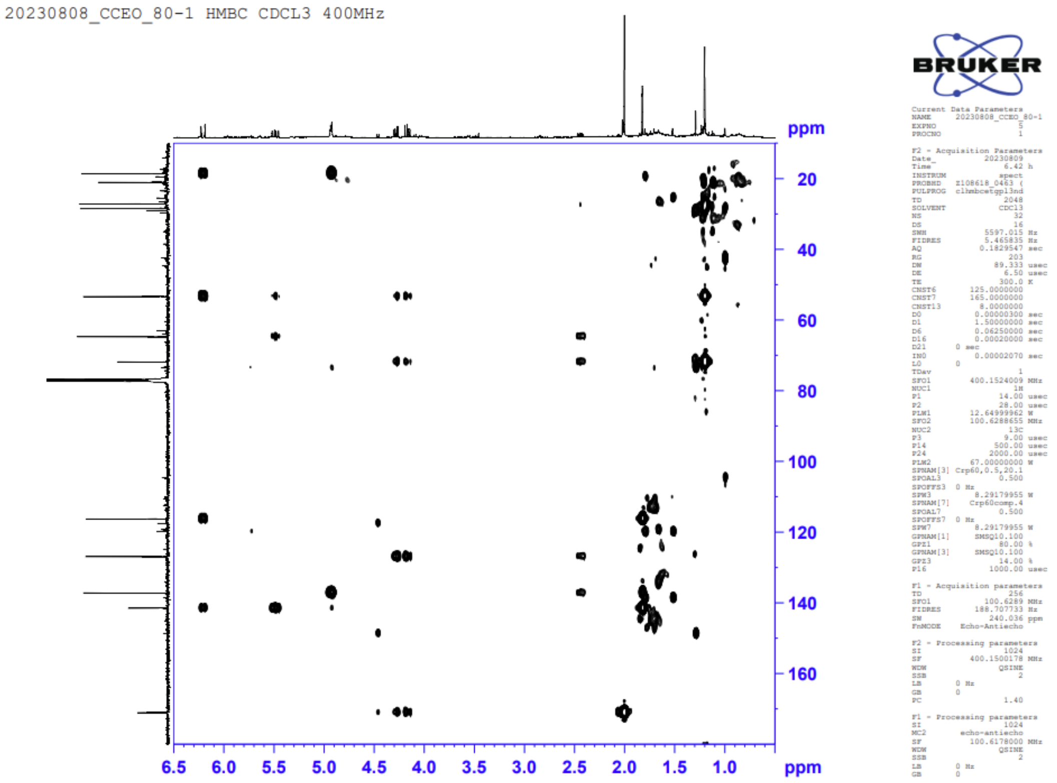

S13. HMBC spectrum of compound 2.

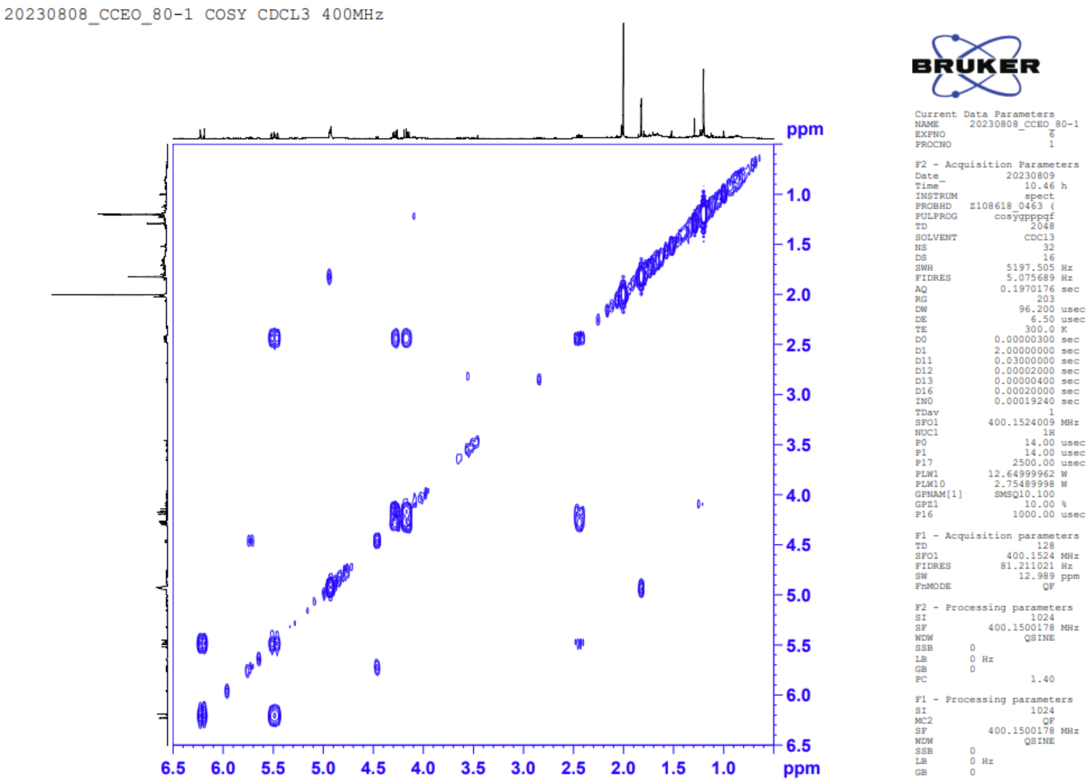

S14. COSY spectrum of compound 2

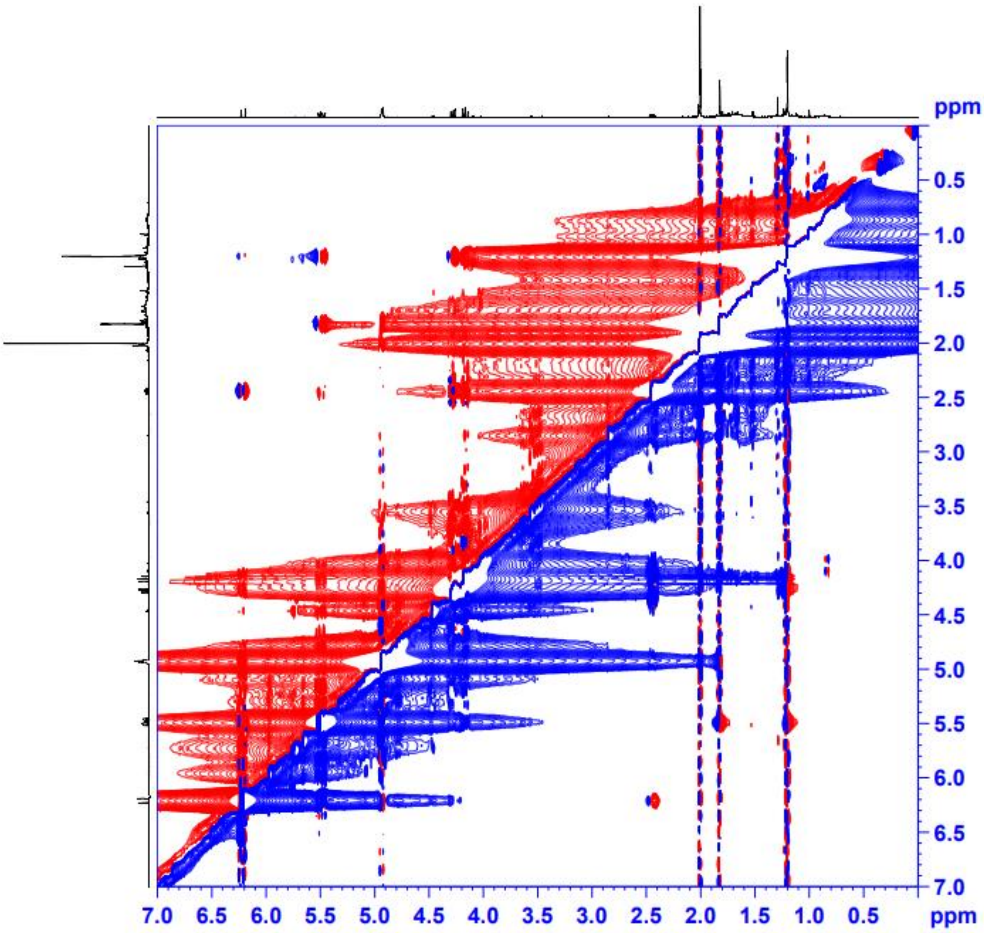

S15. NOSEY spectrum of compound 2.

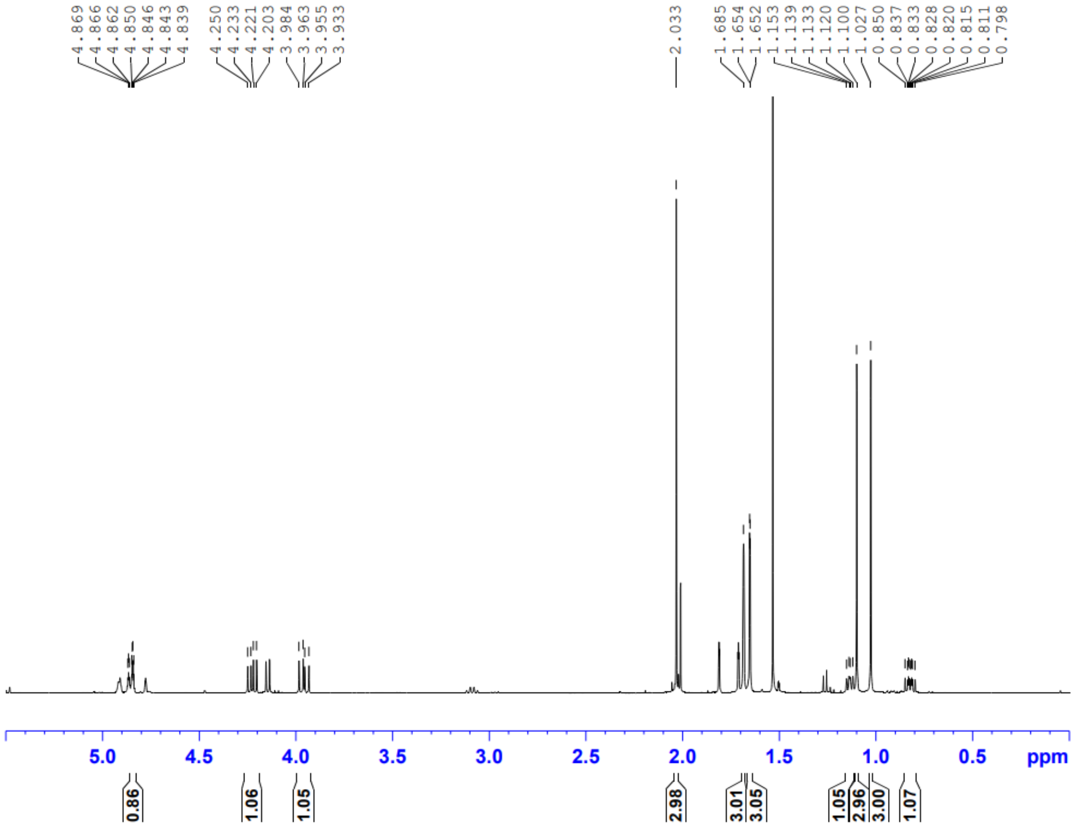

S16. ^{1}H-NMR spectrum of chrysanthemyl acetate ($CDCl_3$, 400 MHz)

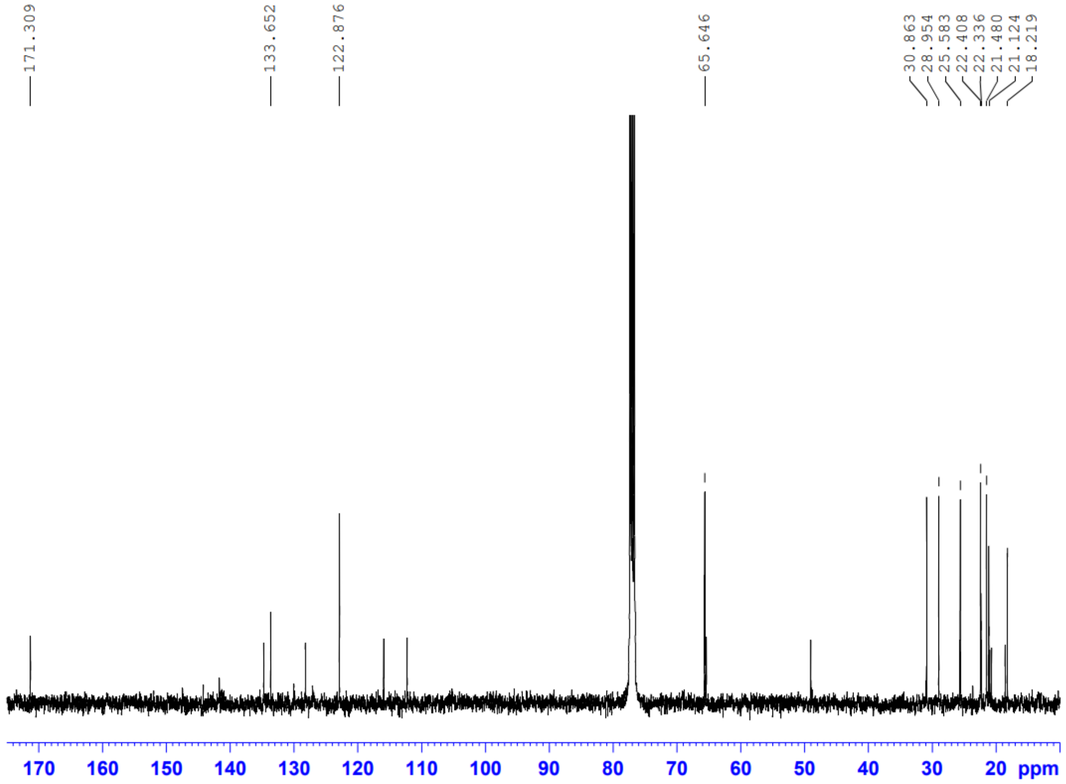

S17. ^{13}C-NMR spectrum of chrysanthemyl acetate ($CDCl_3$, 100 MHz)

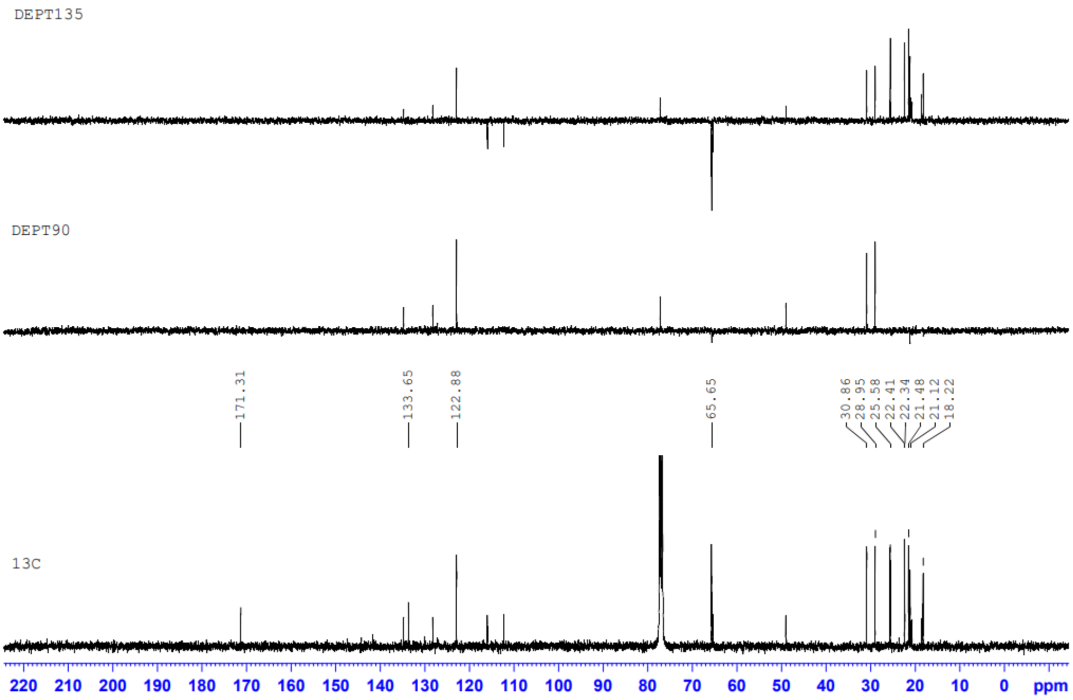

S18. DEPT NMR spectrum of chrysanthemyl acetate ($CDCl_3$, 100 MHz).

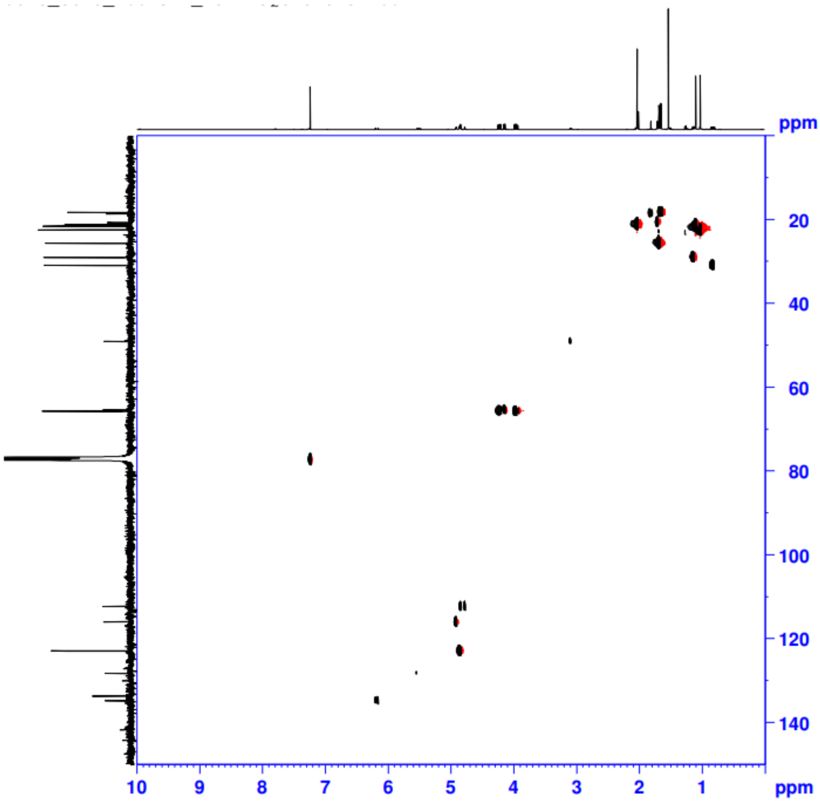

S19. HSQC spectrum of chrysanthemyl acetate.

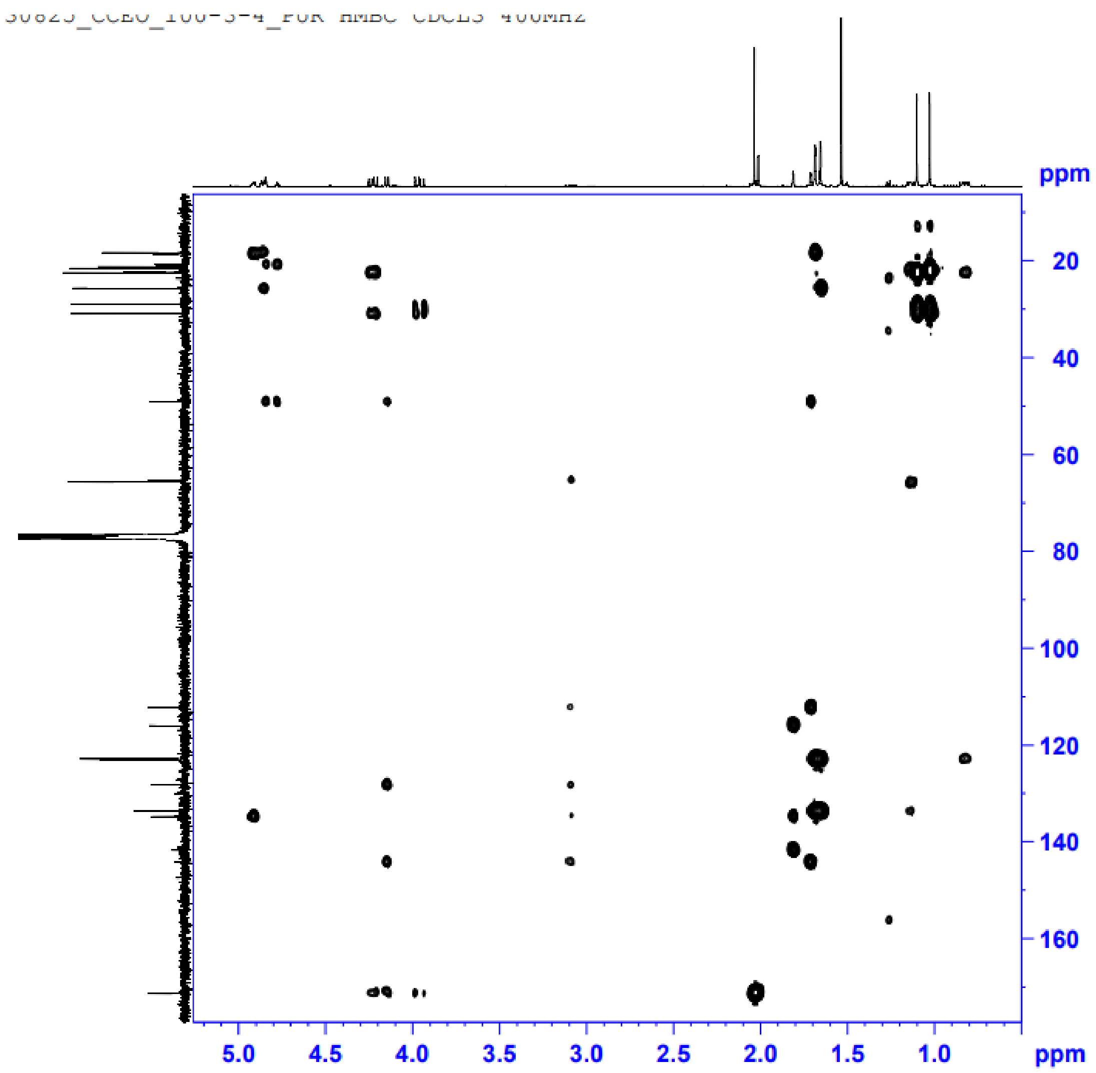

S20. HSQC spectrum of chrysanthemyl acetate.

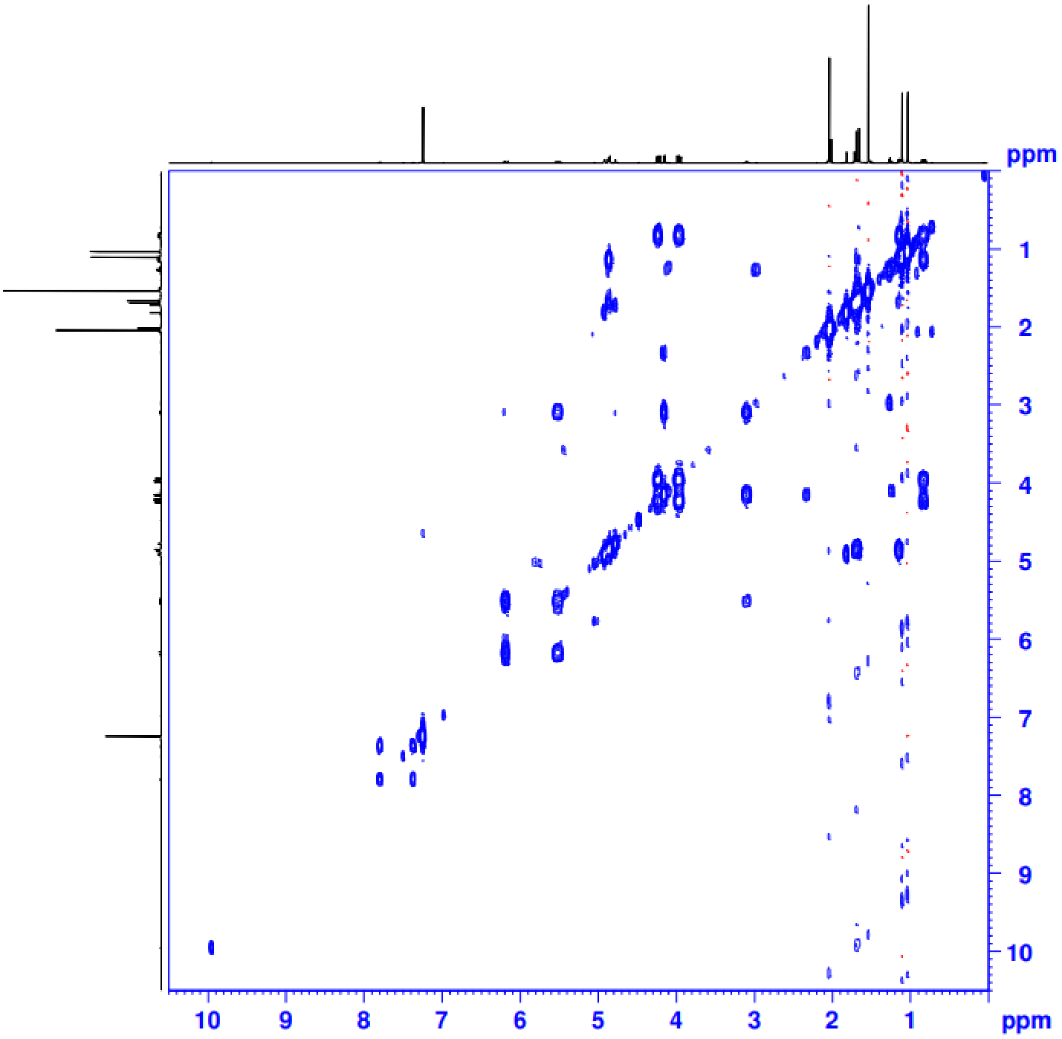

S21. COSY spectrum of chrysanthemyl acetate.

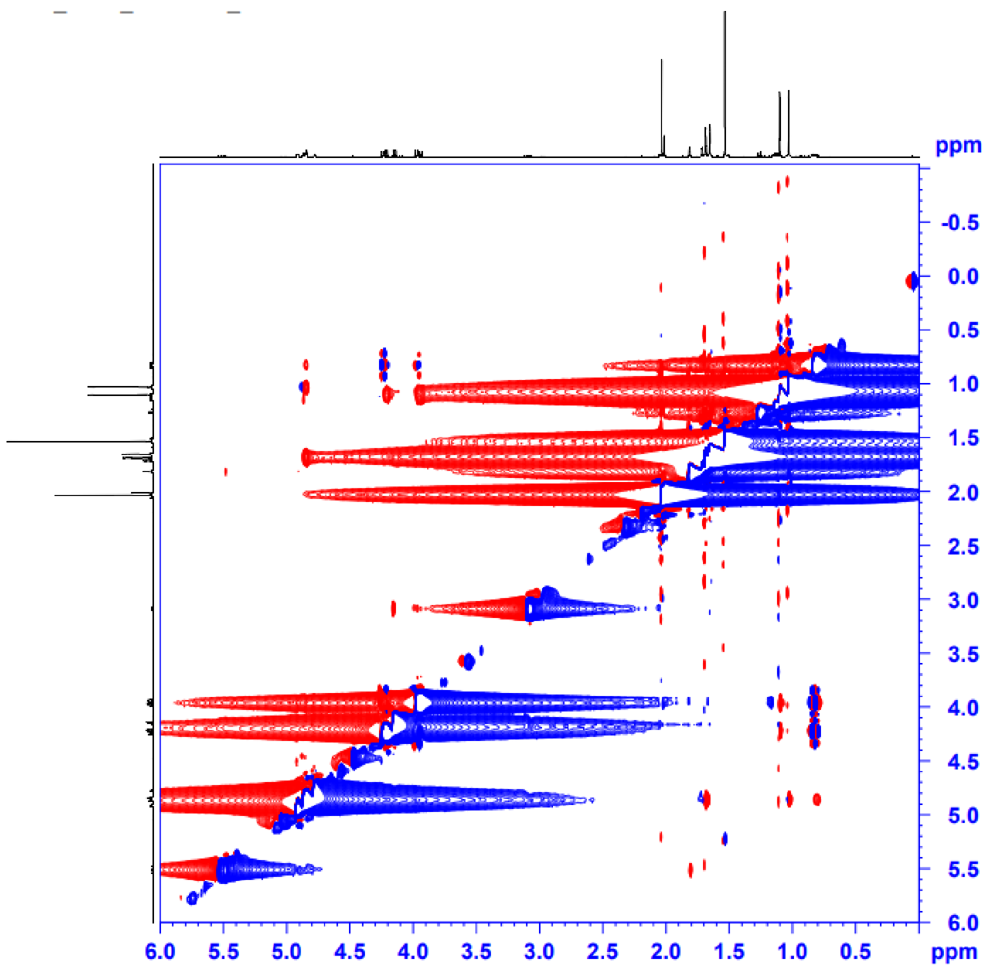

S22. NOESY spectrum of chrysanthemyl acetate.
